# Autoantibodies to protein S may explain rare cases of coagulopathy following COVID-19 vaccination

**DOI:** 10.1038/s41598-024-75514-x

**Published:** 2024-10-18

**Authors:** Ahmet Yalcinkaya, Marco Cavalli, Maribel Aranda-Guillén, Axel Cederholm, Almira Güner, Isabel Rietrae, Hedvig Mildner, Anish Behere, Oskar Eriksson, Laura Gonzalez, Constantin Habimana Mugabo, Anette Johnsson, Tadepally Lakshmikanth, Petter Brodin, Mia Wadelius, Pär Hallberg, Nils Landegren

**Affiliations:** 1 Science for Life Laboratory, Department of Medical Biochemistry and Microbiology, Uppsala University, Uppsala, Sweden; 2 Department of Medical Biochemistry, Faculty of Medicine, Hacettepe University, Ankara, Turkey; 3 Science for Life Laboratory, Department of Immunology, Genetics and Pathology, Uppsala University, Uppsala, Sweden; 4 Department of Medical Sciences, Clinical Pharmacogenomics, Science for Life Laboratory, Uppsala University, Uppsala, Sweden; 5 Department of Medicine (Solna), Center for Molecular Medicine, Karolinska Institutet, Stockholm, Sweden; 6 Department of Immunology, Genetics and Pathology, Uppsala University, Uppsala, Sweden; 7 Unit for Clinical Pediatrics, Department of Women’s and Children’s Health (Solna), Karolinska Institutet, Stockholm, Sweden; 8 Department of Immunology and Inflammation, Imperial College London, London, UK

**Keywords:** COVID-19, Vaccines/adverse effects, Blood coagulation disorders, Autoantibodies, Protein S, Translational research, Autoimmunity, Adaptive immunity, Vaccines

## Abstract

While Coronavirus disease 2019 (COVID-19) vaccines have proven to be both effective and generally safe, rare but severe adverse events following immunization (AEFIs) are described. Autoantibodies to platelet factor-4 are associated with catastrophic thrombotic AEFIs, but comprehensive investigations of other autoantibodies are lacking. We aimed to detect and describe autoantibodies targeting coagulation-related proteins in a population-wide cohort (SWEDEGENE) including AEFIs attributed to COVID-19 vaccines in Sweden. Subjects were recruited from December 2020 to October 2022 and were stratified based on diagnosis and COVID-19 exposure. Screening was carried out in two phases, with a multiplex bead-based assay in the first subset (until September 2021) and with targeted assays for the second (until October 2022). Positivity was defined based on absolute, relative, and biological/technical thresholds. Patients with coagulation-related AEFIs were older and the Vaxzevria vaccine was overrepresented in this group. Two cases had antiphospholipid antibodies but none had PF4 antibodies. We identified six positives for protein S autoantibodies. Protein S concentrations were negatively correlated with autoantibody response in patients with immunoreactivity and functional analysis revealed low protein S activity in three subjects. Our population-wide analysis reveals cases with autoantibodies against protein S which possibly underlie coagulopathic AEFIs.

## Introduction

The COVID-19 pandemic inadvertently became the setting for the first use of various novel-platform vaccines at a population-wide scale^1^. Despite proven safety and efficacy, adverse events following immunization (AEFIs) have been reported in post-authorization studies^2^, some of which were neigh impossible to detect within the scope of expedited clinical trials performed while employing new vaccine technologies^3^. There is limited data concerning mechanisms; however, COVID-19 immunization has been associated with overt autoimmunity^4,5^, which raises valid questions regarding the loss of self-tolerance that could lead to autoimmune-mediated AEFI development^6^.

Thrombotic complications were among the most feared complications of COVID-19. Deficiencies in physiological anticoagulants such as antithrombin III^7^, protein C^8,9^, and protein S^10^, and disruptions in the regulation of coagulation have been implicated in thrombotic events following COVID-19 and other viral diseases^11–13^. In the context of AEFIs, available evidence shows that immune repertoire (ie, antibodies) may incite prothrombotic events^14^. A primary example is antiphospholipid syndrome, a disease in which patients experience increased thrombosis risk due to harboring antibodies against several targets such as apolipoprotein H (ApoH or β2-glycoprotein 1; B2-Gp1) and cardiolipin^15^. These autoantibodies are described as post-viral infection phenomena in pre-pandemic literature^16^ and have been detected in a high proportion of patients with COVID-19-associated coagulability^17,18^–but there are valid doubts regarding their prothrombotic potential^19,20^. A more convincing and direct relationship was defined in patients with vaccine-induced immune thrombotic thrombocytopenia (VITT), in which autoantibodies against platelet factor 4 (PF4) & polyanion (eg, heparin) complexes caused catastrophic thrombotic AEFIs^21–25^. Nonetheless, available data on this topic is based on small studies with limited analyses^22,26^. It is also unclear whether such autoantibodies are persistent or emerge and subside in conjunction with vaccination, which is critical since post-viral antiphospholipid antibodies are often transient and rarely cause coagulopathy^19^.

There is an evident need for larger-scale, comprehensive studies exploring autoantibodies in coagulation-related AEFIs. As such, our goal was to investigate and describe autoantibodies that could underlie coagulation-related AEFIs by leveraging the population-wide data drawn from the SWEDEGENE study, a nationwide cohort created to study underlying factors associated with adverse drug effects in Sweden.

## Methods

### Study design and participants

This exploratory study investigated autoantibodies in patients who had experienced coagulation-related AEFIs secondary to a COVID-19 vaccine, in comparison with patients with other AEFIs, healthy blood donors (BDs), and AEFI cases with COVID-19 exposure. Patients with AEFIs attributed to COVID-19 vaccines were routinely recruited into the SWEDEGENE study (www.swedegene.se). Recruitment followed the standardized SWEDEGENE methodology^27^. Briefly, we contacted and recruited patients reported to the Swedish Medical Products Agency (MPA) due to a suspected AEFI attributed to a COVID-19 vaccine used in Sweden, which included Comirnaty (Tozinameran, BNT162b2; Pfizer), Spikevax (Elasomeran, mRNA-1273; Moderna), and Vaxzevria (Chimpanzee adenovirus Y25, Covishield, ChAdOx1 nCoV-19, AZD1222; AstraZeneca). Based on available literature reporting vaccination data until October 2021, 82.5% of the population in Sweden had been vaccinated with at least one dose, and 75.7–79% of vaccinated individuals had received at least one dose of Comirnaty, while corresponding percentages for Moderna and Vaxzevria were 9.1–14.1% and 5–15.2%, respectively^28–30^. Causality assessment for AEFIs were performed according to World Health Organization (WHO) criteria, as described previously^31^. The first subset of samples analyzed in this study were cases with AEFIs that had occurred between December 2020 and September 2021 and had been reported to the MPA between January 2021 and September 2021. The second subset included cases with AEFIs that had occurred until June 2022 that had been reported to the MPA until October 2022 (including available samples from the first subset). An important change in vaccination schedule during the study period was the discontinuation of Vaxzevria use in Sweden after September 2021. As such, the Vaxzevria-attributed AEFI group in the second subset is comprised of subjects from the first subset and also those who received this vaccine prior to its discontinuation but the MPA was notified of the AEFI after September 2021.

The study was approved by the Swedish Ethics Review Board (ethical permit: #2021-06262-01) and all recruitment/analytical processes conformed to the Declaration of Helsinki. All patients provided informed consent and were at least 18 years of age at the time of recruitment. Clinical data (demographics, medical history, drug treatment history, laboratory data, and ancestry) were recorded. Detailed clinical and event-related information concerning individuals with autoantibody positivity for any antigen were collected by re-examining hospitalization records and discharge reports. Time intervals from vaccination to event (AEFI) and event to sampling were recorded.

Patients with thrombosis, bleeding, thrombocytopenia or myocardial/cerebral infarction were defined as the ‘coagulation-related AEFI’ group. The healthy control group comprised anonymous BDs who had been sampled before the COVID-19 pandemic at Akademiska Sjukhuset, Uppsala University. The ‘other AEFI’ group included patients with anaphylactic reactions, neurological disorders or peri/myocarditis (Supplementary Table 1). To curtail the potential bias of exposure to COVID-19 itself, we extracted a fourth group comprising COVID-19-exposed subjects. This latter group was created based on elevated antibody response to the nucleocapsid antigen (N protein) of SARS-CoV-2, since the vaccines administered to patients would not create reactivity to this antigen. Any subject who had a nucleocapsid response exceeding the mean value of the BD group by 10 standard deviations (SDs) was included in the ‘covid-exposed’ group regardless of AEFI type.

### Analysis subsets

The laboratory analyses were performed in two separate steps since patient recruitment was ongoing throughout the study. The first subset comprised 352 patients, among which 104 were BDs and 248 were from the AEFI cohort (120 coagulation-related AEFI, 90 other AEFI, 38 covid-exposed). These samples underwent antibody screening via a multiplex bead-based assay and confirmation was done by ELISA. The second subset was created by including both newly-received samples (until October 2022) and unthawed samples from the first subset—available for 133 of the subjects (93 of which had coagulation-related AEFI). Ultimately, the second subset comprised 272 individuals, among which 43 were BDs and 229 had AEFIs (186 coagulation-related AEFI, 28 other AEFI, 15 covid-exposed). These samples underwent antibody measurement via in-house and commercial ELISA assays to detect autoantibodies against PF4, PF4-polyanion complexes (PF4C) and antiphospholipid antibodies (ApoH, cardiolipin, and screening). The ‘screening’ refers to the use of the Human Phospholipid Screen IgG/IgM ELISA kit, detailed later on in the text.

### Definitions for autoantibody positivity

For the first subset of patients, autoantibody response with the bead-based assay was deemed positive if a particular sample fulfilled three strict criteria: (1) absolute response exceeded the BD mean for that particular antigen by at least 10 SDs, (2) relative response (sample-to-BD mean ratio) exceeded a 10-fold threshold for each antigen, and (3) absolute response was at least 1000 arbitrary units (AUs). Samples fulfilling these criteria were reanalyzed via ELISA to demonstrate elevated response relative to randomly-selected BDs and samples from patients with myocarditis. For the second subset, antibody responses were defined to be positive if they exceeded thresholds created by simultaneously-measured biological samples with known autoantibody positivity for said antigen (or exceeding thresholds defined by commercial kits). When necessary, functional analyses were also performed to confirm the physiological impact of autoantibodies.

### Sampling and autoantibody detection

#### Sampling process

Blood samples were drawn at the patient’s nearest health-care facility (heparinized samples, centrifuged at 1500×g, 10 min, 4 °C), and the resultant plasma was aliquoted and transferred to Uppsala University (stored at −70 °C).

#### Bead-based immunoassay

A multiplex bead-based immunoassay was performed to detect autoantibodies against multiple target antigens, including the following coagulation-related proteins: Factor V, Protein S, Protein C, Prothrombin, PF4, ApoH (B2-Gp1), and Antithrombin III. Sample detection was confirmed by anti-human IgG response. Antibody response against the Epstein-Barr virus nuclear antigen 1 (EBNA1) was also determined to confirm antibody detection and demonstrate the detection of variabilities in reactivity. Additionally, vaccine response and SARS-CoV-2 exposure were examined by measurement of responses against the Spike (S protein), receptor binding domain (RBD), and nucleocapsid (N protein) antigens.

The first step in the established protocol was the creation of beads coupled to targeted antigens, as detailed previously^32–34^. Magnetic beads (MagPlex®, Luminex) were coupled with commercial, full-length target proteins by use of an AnteoTech activation kit (A-LMPAKMM-10). For each antigen, the protein-to-bead concentration was 3 µg/1.5 × 10^6^ beads. Samples (1 µl) were then diluted 1:250 through a 2-step process: 1:25 in phosphate-buffered saline (PBS) and then 1:10 in PBS containing 0.05% Tween-20, 3% bovine serum albumin (BSA) and 5% non-fat milk. The resultant working samples (250 µl total volume) were incubated with 5 µl of the bead suspension (2 h at room temperature) under slight agitation (orbital shaker at 650 revolutions per minute–RPM). After magnetization and 3 wash cycles (0.05% Tween-20 in PBS), resuspension was performed in 50 µl of 0.2% paraformaldehyde for 10 min, followed by another 3-cycle wash. Secondary antibodies were incubated for 30 min [F(ab)'2-Goat anti-Human IgG Fc; H10104, Invitrogen]. Detection was carried out with a FlexMap 3D analyzer.

#### In-house ELISA

ELISAs were developed for the detection of IgG/A/M antibodies against the following molecules: PF4, B2-Gp1, and cardiolipin. All ELISAs were developed with clear, high-binding, half-area 96-well plates (734-1624, Corning, VWR). For protein coating, the final protocol was to obtain 1–2 µg/ml protein diluted in PBS containing 0.01% BSA. Fifty µl of protein solution was added to each well, the plate was sealed, and coating was performed overnight at 4 °C. For cardiolipin, the protocol was to obtain 10 µg/ml cardiolipin concentration in 50 µl ethanol (initial purity 99.5%) and the plate was left unsealed at 4 °C overnight for complete evaporation. In the event that complete dryness was not observed on the following day, the plate was left at room temperature for up to 30 min before proceeding with the assay.

The washing buffer was PBS with 0.1% Tween-20, and plate washing was performed in a standard fashion with 130 µl of buffer (5 times). Plates were blocked for 2 h at room temperature using 2% BSA in PBS with 0.01% Tween-20 (55 µl). Samples and positive controls were diluted with PBS (1:2000) in a 2-step process (1:20 then 1:100) before being immediately transferred to wells for incubation (50 µl, 1.5 h at room temperature) with slight agitation achieved on an orbital shaker set to 200 RPM. Secondary antibodies were added at a volume of 50 µl for 1 h (diluted at 1:10000). Different secondary antibodies were used to identify Ig types (IgG/A/M, IgG, and IgM; Invitrogen A18847, A18805, and 31415). Color development was achieved by 5–10 min of 3, 3', 5, 5' tetramethylbenzidine (TMB) incubation. The reaction was stopped with 40 µl of 0.2 M H_2_SO_4_, and the optical density was recorded at 450 nm (Magellan, TECAN).

#### Commercial assays

To detect antibodies against PF4-polyanion complexes (PF4C), we used the Lifecodes PF4 Enhanced assay (X-HAT45G, Immucor), which is used for diagnostic purposes in patients with heparin-induced thrombocytopenia and shows excellent sensitivity for VITT^35^. An additional step to confirm antiphospholipid antibodies (such as B2-Gp1 and cardiolipin) was performed on samples with relatively elevated levels in the bead-based assay or in-house ELISA, by using a commercial screening kit capable of detecting IgG/M autoantibodies (Arigo Biolaboratories, ARG80405, Human Phospholipid Screen IgG/IgM). Protein S levels were measured using an ELISA kit (Novus Biologicals, NBP2-60585, Lot# 101802311), with calculation performed via four-parameter logistic regression.

#### Confirmatory analyses

For the confirmation of protein S autoantibodies, we employed a separate optimized ELISA on the following samples: the 6 patients with the highest responses in the bead-based assay (confirmation subgroup), 8 randomly-selected patients with myocarditis, and 8 randomly-selected BDs. Protein S was coated at 1.6 µg/ml in PBS, blocking buffer was 3% BSA in PBS with 0.05% Tween-20, samples were added to wells with 1:250 final dilution, and secondary antibody was diluted 1:8000. All other steps of the protocol were the same as described above (in-house ELISA).

Protein S activity was also tested through a manual method, utilizing a functional assay (ACTICLOT Protein S, BioMedica Diagnostics) which outputs a percentage-wise protein S activity value based on sample clotting time. Since the blood samples from patients with AEFIs were collected in heparinized tubes, the assay was not readily applicable to these samples (demonstrated to be working with EDTA and citrated plasma). We utilized protein A and protein G magnetic beads to purify IgG from the heparinized plasma of the 8 patients with the highest immunoreactivity to protein S. These were mixed with pooled EDTA plasma to create assayable samples (Supplementary methods). Time until clot development was kept manually. The reference range for normal protein S activity was 55–160%. The decision to include 8 subjects with available samples in this analysis (instead of only the 6 subjects defined to have positivity) was made to improve data comprehensiveness based on the fact that the bead-based results obtained for these additional patients were very close to the thresholds set for positivity.

### Statistics

All data were entered into SPSS (.sav) databases which were imported into Rstudio software using the “haven” and “sjlabelled” packages. Missing data were not imputed and were excluded from analyses. For data visualization, we used the “ggplot2” and “pheatmap” packages for R (version 4.3.0–“Already Tomorrow”; Rstudio release “Ocean Storm”, 2024-01-28)^36^. To obtain data summaries (descriptives) and perform statistical analyses, we used the SPSS v25.0 (IBM, NY, USA) software. Numerical data were summarized in the form of mean ± SD, while nominal and ordinal data were summarized with absolute (*n*) and relative frequencies (%). For all categorical variables, we used appropriate Chi-square tests or the Fisher’s Exact test to test for differences in relative distribution between groups. For numerical variables, histograms and Q-Q plots were used to assess normality of distribution, supplemented with the Kolmogorov-Smirnov (Lilliefors correction) test to exclude normality. Comparison of numerical variables between two independent groups was performed with the Mann-Whitney U test, while > 2-group comparisons were performed with one-way ANOVA (parametric) or the Kruskal–Wallis test (non-parametric), with Bonferroni correction used for pairwise analysis. The effect sizes of directional relationships between continuous variables were analyzed by calculating the Pearson correlation coefficient (r).

## Results

**Fig. 1:**
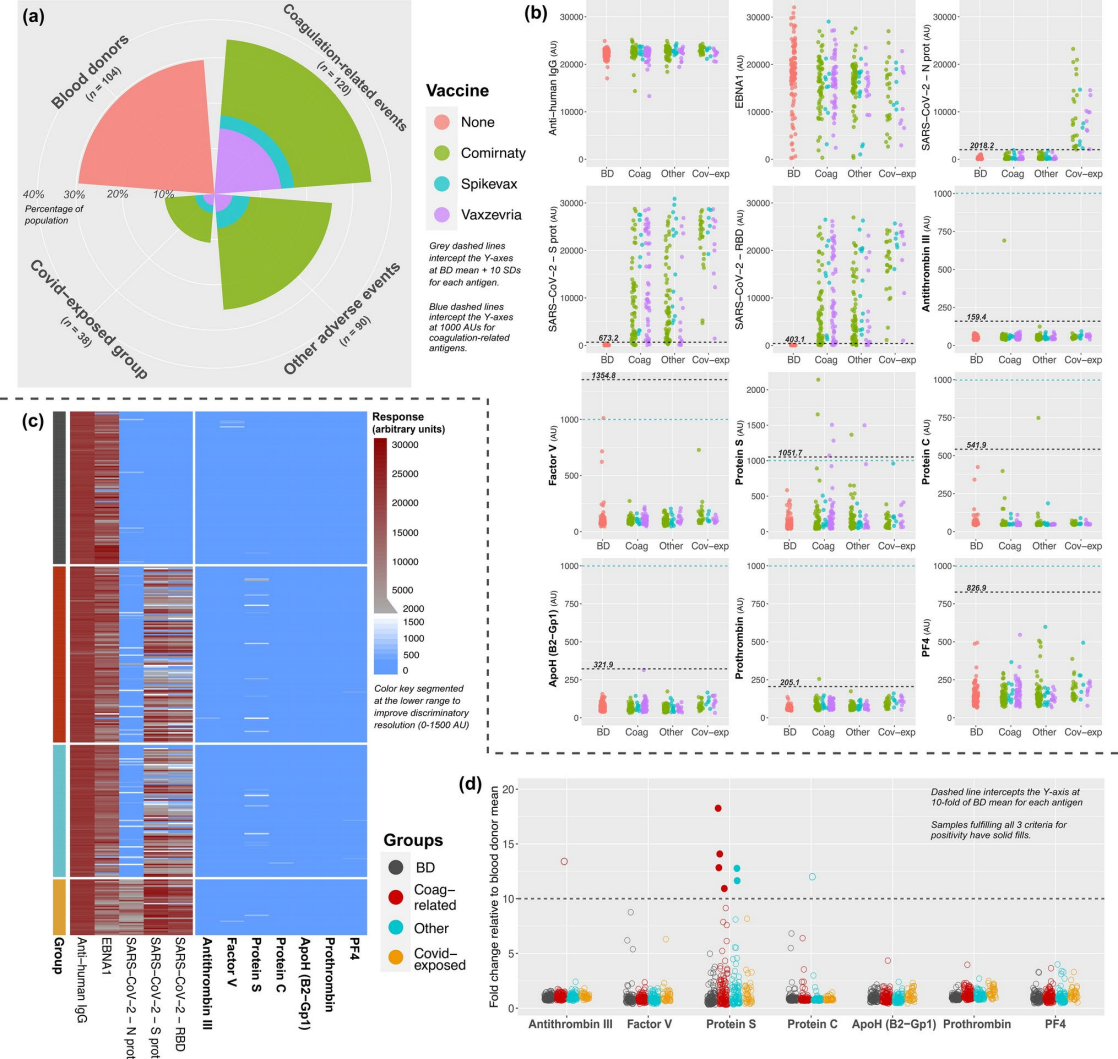
Autoantibody data and positivity thresholds for bead-based screening of the first subset. Coxcomb chart (**a**) illustrates group sizes and vaccine types. Absolute-value scatterplots (**b**) are used to compare autoantibody responses in the analyzed groups, stratified based on vaccine type. Grey and blue dashed lines define the ‘BD mean + 10 SDs’ and the arbitrary unit threshold (1000 AUs) for positivity. Heatmap (**c**) illustrates bead-based responses in the study groups on a segmented color scale (see key). Fold-change scatterplot (**d**) visualizes samples with respect to the 10-fold relative-elevation threshold used as a criterion for positivity. Solid-filled points show the six samples that met all three criteria for Protein S autoantibody positivity. Abbreviations: AU: arbitrary unit, BD: blood donor, AEFI: adverse events following immunization, Coag: coagulation-related, Cov-exp: COVID-19-exposed AEFI group, ApoH: Apolipoprotein H, B2-Gp1: β2-glycoprotein 1, PF4: platelet factor 4, AU: arbitrary unit, IgG: immunoglobulin G, EBNA1: Epstein-Barr virus nuclear antigen 1, S prot: spike protein, RBD: receptor binding domain, N prot: nucleocapsid protein.

The first SWEDEGENE subset received for analysis comprised 352 individuals, including 104 BDs, 120 coagulation-related AEFIs, 90 other AEFIs, and 38 covid-exposed patients with AEFIs. Among the 248 patients with an AEFI, 145 (58.5%) received Comirnaty, 29 (11.7%) received Spikevax, and 74 (29.8%) received Vaxzevria. Sex distribution was similar in all AEFI subgroups (Pearson chi-square, *p* = 0.726); however, patients in the coagulation-related group were significantly older than patients in other groups (Kruskal–Wallis, *p* < 0.001). Vaxzevria recipients were overrepresented in the coagulation-related group (42.5%) compared to other groups (15–25%). Vaccination-to-event and event-to-sampling times were significantly longer in the coagulation-related group compared to the other groups (*p* < 0.001 and *p* = 0.029, respectively). Anti-human IgG response was similar in all groups (one-way ANOVA, *p* = 0.072), EBNA1 demonstrated anticipated variations, and SARS-CoV-2 responses were in-line with vaccination and exposure (Table 1; Fig. 1a, b).

**Table 1:** Group distribution and summary of characteristics in the first subset.

	Blood donors (n = 104)	Coagulation-related (n = 120)	Other AEFI (n = 90)	Covid-exposed (n = 38)	*P*
Sex, n (%)					
Female	54 (51.9%)	64 (53.3%)	53 (58.9%)	19 (50%)	0.726
Male	50 (48.1%)	56 (46.7%)	37 (41.1%)	19 (50%)	
Age, years	52 ± 12.4	63.3 ± 16†	49.1 ± 18.2	50.4 ± 13.8	**< 0.001**
Vaccine, n (%)					
None	104 (100%)	0 (0%)	0 (0%)	0 (0%)	0.001*
Comirnaty	0 (0%)	59 (49.2%)	63 (70%)	23 (60.5%)	
Spikevax	0 (0%)	10 (8.3%)	13 (14.4%)	6 (15.8%)	
Vaxzevria	0 (0%)	51 (42.5%)	14 (15.6%)	9 (23.7%)	
Dose count, n (%)					
One dose	N/A	93 (77.5%)	69 (76.7%)	23 (60.5%)	0.095*
Two doses	N/A	27 (22.5%)	21 (23.3%)	15 (39.5%)	
Time from vaccination to event, days	N/A	13.99 ± 9.47†	6.82 ± 10.77	9.75 ± 13.13	< 0.001*
Time from event to sampling, days	N/A	165.47 ± 63.05	150.93 ± 65.6	139 ± 61.86	0.029*‡
Anti-human IgG	22274 ± 961	22404 ± 1516	22653 ± 1081	22731 ± 861	0.072
Anti-EBNA1	17895 ± 6832§	16049 ± 5677	15739 ± 5031	13690 ± 6383	**< 0.001**
Anti-SARS-CoV-2\-N prot	168.5 ± 185	244.8 ± 365.5	293.7 ± 395.7	8910 ± 5982**||**	< 0.001*
Anti-SARS-CoV-2-S prot	77.13 ± 59.61	11598 ± 9383	10405 ± 9254	21902 ± 6698**||**	< 0.001*
Anti-SARS-CoV-2-RBD	59.61 ± 34.35	9128 ± 8205	7481 ± 7648	17914 ± 6435**||**	< 0.001*

Bold values indicate statistical significance.

AEFI: adverse events following immunization, N/A: not applicable, IgG: immunoglobulin G, EBNA1: Epstein–Barr nuclear antigen 1, SARS-CoV-2: Severe acute respiratory syndrome coronavirus 2, N prot: nucleocapsid protein, S prot: spike protein, RBD: receptor-binding domain.

(*) The BD group was excluded from statistical analyses.

(†) Coagulation group values significantly higher than other groups (Kruskal–Wallis, Bonferroni-corrected *p* < 0.001, for all pairwise).

(‡) All three pairwise comparisons are non-significant (Bonferroni correction).

(§) Blood donor values significantly higher than other groups (Kruskal–Wallis, Bonferroni-corrected *p* = 0.023 vs. Coagulation, *p* = 0.009 vs. Other AEFI, *p* = 0.001 vs. Covid-exposed).

(||) Covid-exposed values significantly higher than other groups (Kruskal–Wallis, Bonferroni-corrected *p* < 0.001, for all pairwise).

The multiplex assay identified 10 samples in which autoantibodies exceeded the absolute value threshold (BD mean + 10 SDs) for antithrombin III (n = 1), protein S (n = 7), protein C (n = 1), and prothrombin (n = 1) (Fig. 1b,c). After subsequent analysis for relative increase (> 10-fold), we defined a total of 8 elevated autoantibody responses (one for antithrombin III, 6 for protein S, one for protein C) in 7 patients (Fig. 1d). Since responses exceeded 1000 AUs for only those with reactivity to protein S, we concluded that only these 6 cases (2.42% of 248 AEFI subjects) were positive for autoantibodies. Increased immunoreactivity to protein S was confirmed in all 6 cases via optimized ELISA (Supplementary Fig. 1). Event-to-sampling interval ranged from 50 to 238 days among these subjects, indicating long-term detectability and supporting the notion that these antibodies were persistent.

**Fig. 2:**
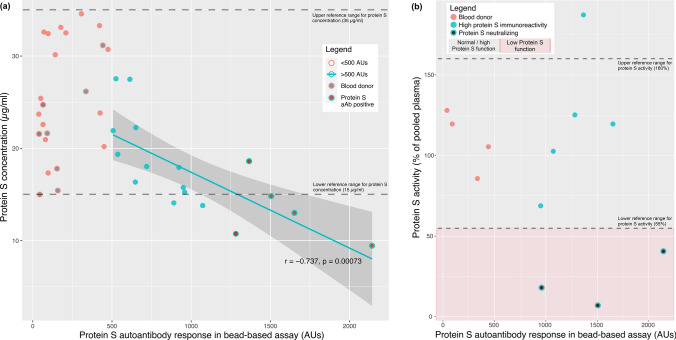
Confirmatory testing results for protein S autoantibodies. Scatterplot (**a**) shows protein S concentration and autoantibody response in different subgroups as stratified by the legend as well as the correlation curve for subjects with > 500 AU response to protein S in the bead-based assay. Correlation coefficient (Pearson r) and *p* value refers to all subjects with response exceeding 500 AUs. Protein S activity results in relation with protein S concentrations (**b**) reveals three patients with reduced protein S activity (< 55% of pooled plasma) and one patient with increased activity. Note: one patient with protein S autoantibody positivity did not undergo confirmatory analyses due to unavailability of sample. Abbreviations: AU: arbitrary unit, aAb: autoantibody.

To understand the functional impact of protein S autoantibodies, we sought to quantify circulatory protein S levels and perform functional analysis. Protein S concentrations were analyzed in a subgroup comprised of 40 subjects (18 with > 500 AU antibody response, and 22 randomly-selected BDs and AEFI patients). The > 500 AU group and the randomly-selected subjects were similar in terms of age (Mann-Whitney U, *p* = 0.100) and sex distribution (*p* = 0.262). Further comparison between the antibody-positive, antibody-negative, and BD subjects in this subgroup again showed lack of significant differences in terms of age (Kruskal–Wallis, *p* = 0.423) and sex distribution (p = 0.355). Among the 18 samples with antibody response exceeding 500 AUs, we detected a strong negative correlation between protein S concentration and antibody response (r = −0.737, *p* < 0.001). No correlation was present in the randomly-selected subset of 22 subjects (r = 0.352, *p* = 0.100) (Fig. 2a). Functional analysis of protein S in 8 samples with the highest immunoreactivity to protein S revealed three subjects with activity percentages below the 55% threshold (Fig. 2b). Two of these patients had suffered from coagulation-related AEFI and fulfilled positivity criteria applied for the initial bead-based assay.

**Fig. 3:**
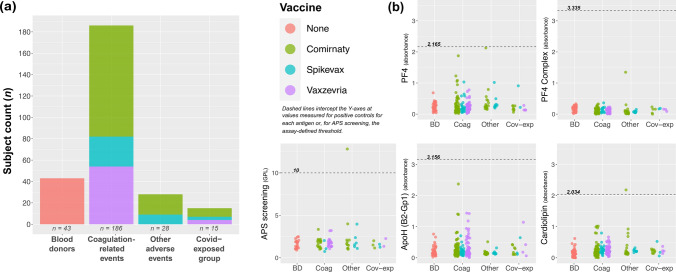
Autoantibody results and thresholds for antigens examined in the second subset. Stacked bar chart (**a**) shows group sizes by vaccine type. Scatterplots (**b**) show measurement results for PF4, PF4 complex, APS screening, ApoH (B2-Gp1), and cardiolipin. Dashed lines are results obtained from positive controls, or in the case of APS screening, the positivity threshold defined by the kit (in GPL units; 1 GPL is accepted to equate to 1 µg of relevant antibody). Abbreviations: BD: blood donor, AEFI: adverse events following immunization, Coag: coagulation-related, Cov-exp: COVID-19-exposed AEFI group, ApoH: Apolipoprotein H, B2-Gp1: β2-glycoprotein 1, PF4: platelet factor 4, APS antiphospholipid syndrome, MPL: IgM phospholipid units, GPL: IgG phospholipid units.

The second patient subset comprised 272 subjects (43 BDs, 186 coagulation-related AEFIs, 28 other AEFIs, and 15 covid-exposed AEFIs). Vaccine distribution among the 229 AEFI patients was: 131 (57.2%) Comirnaty, 40 (17.5%) Spikevax, and 63 (25.3%) Vaxzevria. The sexes were again similarly distributed in all groups (*p* = 0.372). The coagulation-related subgroup was older compared to the BD (Kruskal–Wallis, Bonferroni-corrected *p* < 0.001) and the other AEFI subgroups (*p* < 0.001), but not the covid-exposed group (*p* = 0.205). Vaxzevria recipients in the coagulation-related group demonstrated a markedly lower frequency in the second (29%) compared to the first subset (42.5%), likely owing to the discontinuation of Vaxzevria use. Vaccination-to-event time was similar in the three AEFI groups, while event-to-sampling time was significantly longer in the other AEFI group (*p* < 0.001) (Table 2; Fig. 3a). There were no patients with autoantibodies against PF4 or PF4C. Only two samples with positivity for antiphospholipid antibodies were detected (both IgG-type); however, neither of these patients had experienced coagulation-related AEFI (Fig. 3b).

**Table 2:** Group distribution and summary of characteristics in the second subset.

	Blood donors (n = 43)	Coagulation-related (n = 186)	Other AEFI (n = 28)	Covid-exposed (n = 15)	*P*
Sex, n (%)					
Female	26 (60.5%)	94 (50.5%)	11 (39.3%)	8 (50%)	0.372
Male	17 (39.5%)	92 (49.5%)	17 (60.7%)	7 (50%)	
Age, years	47.4 ± 13.7	62.4 ± 15.8†	46 ± 12	54.9 ± 9.8	**< 0.001**
Vaccine, n (%)					
None	43 (100%)	0 (0%)	0 (0%)	0 (0%)	0.001*
Comirnaty	0 (0%)	104 (55.9%)	19 (67.9%)	8 (53.3%)	
Spikevax	0 (0%)	28 (15.1%)	9 (32.1%)	3 (20%)	
Vaxzevria	0 (0%)	54 (29%)	0 (0%)	4 (26.7%)	
Dose count, n (%)					
One dose	N/A	105 (56.5%)	6 (21.4%)	7 (46.7%)	0.002*
Two doses	N/A	51 (27.4%)	12 (42.9%)	8 (53.3%)	
Three doses	N/A	25 (13.4%)	7 (25%)	0 (0%)	
Four doses	N/A	5 (2.7%)	3 (10.7%)	0 (0%)	
Time from vaccination to event, days	N/A	19.77 ± 20.24	24.04 ± 40.8	13.8 ± 9.37	0.605*
Time from event to sampling, days	N/A	227.56 ± 119.16	338.89 ± 108.47‡	174.4 ± 53.83	< 0.001*

Bold values indicate statistical significance.

Abbreviations are explained in Table 1.

(*) The BD group was excluded from statistical analyses.

(†) Coagulation group values significantly higher than BD and other AEFI (Kruskal–Wallis, Bonferroni-corrected *p* < 0.001, for both).

(‡) Other AEFI group values significantly higher than the Coagulation (*p* = 0.001) and Covid-exposed (*p* < 0.001) groups (Bonferroni correction).

The diagnoses, time frames, and the clinical and event-related characteristics of autoantibody-positive patients (n = 8) are detailed in Table 3. Additionally, protein S concentration and activity levels are reported for patients positive for protein S autoantibodies.

**Table 3:** Detailed clinical findings and progression of individuals with autoantibody positivity.

#	Autoantibody and related data	Sex, age	Exact diagnosis	Vaccination-to-event time	Event-to-sampling time	Vaccine, dose	Presentation and findings	Clinical history and management
#1	Protein S, Conc: 9.46 ug/ml, Activity: 40.6% (low)	Female, 55	Coagulation-related: Thrombophlebitis	30 days	92 days	Comirnaty, second	Post-vax day 30: Hospitalized with thrombophlebitis and varicose veins, without trauma history.	Superficial venous insufficiency history. Used supplements containing magnesium and vitamins B and D within prior 3 months. Negative for COVID-19 (anti-N protein: 140 AUs). Discharged following supportive treatment.
#2	Protein S, Conc: 13.01 ug/ml, Activity: 119.6%	Female, 24	Coagulation-related: Thrombocytopenia	21 days	50 days	Comirnaty, second	Post-vax day 20: Excessive menstrual bleeding. Purpura developed after suffering from an unusually large bruise in a contact sport. Post-vax day 21: Severe thrombocytopenia without thrombotic complications. Platelets: 6000/µl, Leukocytes: 2700/µl, Hemoglobin: 13.0 g/dl, Erythrocytes: 4.2 × 10^6^/ml	No comorbidities, used paracetamol within prior 3 months. Negative for COVID-19 (anti-N protein: 128.5 AUs). Treated with prednisolone 1 mg/kg/day. Eventually tapered down to 10 mg/day and terminated after return to normal platelet counts. Discharged without other remarks.
#3	Protein S, Conc: 14.83 ug/ml, Activity: 6.76% (low)	Male, 78	Coagulation-related: Pulmonary emboli	19 days	101 days	Vaxzevria, first	Post-vax day 29: Admitted with poor general condition and fatigue. CRP: 70, Leukocytes: 10500/µl, Hb 12.5 g/dl, D-dimer: 3.1. Troponin I: 17 ng/L, NTproBNP: 467. Thorax CT confirmed emboli in conjunction with symptomatology. Imaging studies revealed pulmonary embolism, other potential causes of pulmonary emboli were ruled out.	Comorbidities: high blood pressure, myocardial infarct, sick sinus syndrome (pacemaker), hypercholesterolemia. Used candesartan, hydrochlorothiazide, bisoprolol, acetylsalicylic acid, simvastatin and vitamin D within prior 3 months. Negative for COVID-19 (anti-N protein: 163 AUs). Started on Eliquis therapy planned for 6 months. Routine management, recovering at last follow-up visit.
#4	Protein S, Concentration and activity not measured due to unavailability	Male, 71	Other AEFI: Guillain-Barré	13 days	235 days	Vaxzevria, first	Symptoms started with paresthesia in the hands and feet. Over few days, disease progressed to weakness in both arms and legs. Autonomic dysfunction and symptoms of cranial nerve involvement were noted. Neurophysiological findings, cerebrospinal fluid results, and laboratory analyses were consistent with Guillain-Barré syndrome.	Comorbidities: diabetes mellitus type 2, hypertension, surgery for prostate cancer (3 years prior). Negative for COVID-19 (anti-N protein: 60 AUs). Treated with plasma exchange and intravenous immunoglobulin (IVIG) with moderate efficacy.
#5	Protein S, Conc: 18.61 ug/ml, Activity: 187.2% (high)	Female, 77	Other AEFI: Vestibular neuritis	4 days	184 days	Comirnaty, first	Post-vax day 4: admitted to hospital due to poor general condition. Described dizziness since the same morning. Stroke ruled out. Post-vax day 5: movements exceedingly limited due to vertigo. Brain MRI normal. Diagnosed with left-sided vestibular neuritis.	Comorbidities: high blood pressure, hyperlipidemia, history of benign paroxysmal positional vertigo. Takes metoprolol, lercanidipine. Smoker (10 cigarettes per day). Alcohol use 6 days per week. Negative for COVID-19 (anti-N protein: 74.5 AUs). Treated with methylprednisolone, discharged on post-vax day 9 following significant improvement.
#6	Protein S, Conc: 10.75 ug/ml, Activity: 125.18%	Female, 73	Coagulation-related: Pulmonary emboli and Cerebral infarct	2 days	238 days	Vaxzevria, first	Post-vax day 3: Patient found unconscious and was presumed to be in such state for around 2 days. Electrocardiography normal. Thorax CT showed pulmonary embolism. Brain CT revealed a small infarct in the left thalamus.	Comorbidities: Depression, rheumatoid arthritis, goiter, gastroesophageal reflux, hypertension. Uses lithium and omeprazole. Negative for COVID-19 (anti-N protein: 186 AUs). Treated with Metoprolol for sinus tachycardia, Eliquis for pulmonary emboli, and Atorvastatin was begun for stroke. Improvement noted by post-vax day 50, except for recurring headaches.
#7	Anti-phospholipid, IgG-type	Male, 26	Other AEFI: Myocarditis	4 days	412 days	Comirnaty, second	Post-vax day 1: Fatigue, fever, asthenia, and headache. Day 3: Minor chest pain and syncope episode. Day 4: Admitted for chest pain radiating to the left arm. Electrocardiography was normal, but troponin I > 12674 ng/L, CRP: 11 mg/L, leukocytes: 8900/µl, NTproBNP: 388. Other blood counts normal. Transthoracic echo and heart MRI confirmed myocarditis without pericarditis.	No comorbidities. Used ibuprofen, loperamide and floxacillin within prior 3 months. Negative history for COVID-19 (anti-N protein not measured). Managed with supportive treatment. Complete recovery noted on post-vax day 52.
#8	Anti-phospholipid, IgG-type	Male, 19	Other AEFI: Myocarditis	53 days	360 days	Comirnaty, second	Post-vax day 43: Flu-like symptoms Post-vax day 53: chest pain and hospital admission. Electrocardiography normal. Max troponin I: 47000 ng/L, NTproBNP: 70, CRP: 2.2, no other abnormalities. Electrocardiogram normal. Myocarditis confirmed with MRI.	Comorbidity: asthma. Regular use of inhaler (Budesonide + formoterol). Negative history for COVID-19 (anti-N protein not measured). Discharged after complete recovery on post-vax day 104. Monitored with Telemedicine after discharge, reported returning to normal daily life without any complaints.

## Discussion

COVID-19 infection is well-understood to create a prothrombotic state^11,37^, and although vaccination greatly reduces the likelihood of thrombosis^38^, a number of studies have shown COVID-19 vaccine-triggered thrombotic events^39^. Our data obtained from the nationwide SWEDEGENE cohort confirmed that the Vaxzevria vaccine was overrepresented in our initial subset of patients who had been vaccinated before the use of this vaccine was restricted due to hypercoagulability concerns. PF4 autoantibodies, which are well understood to cause catastrophic thrombosis among certain vaccine recipients, appear to be exceedingly rare. The comprehensive screening to detect autoantibodies targeting coagulation-related proteins revealed increased reactivity towards protein S in 6 individuals who fulfilled stringent criteria for positivity, and the event-to-sampling times indicate that these autoantibodies could be detected long after the events –suggesting persistence. The negative correlation between protein S level–autoantibody response and the low protein S activity among 3 patients (2 of whom had coagulation-related AEFI) further support our interpretation that protein S autoantibodies could explain rare cases of coagulopathy following COVID-19 vaccination.

Autoantibodies are increasingly being studied to understand their mechanistic roles in various diseases, revealing many pathologies that are either caused or worsened by the loss of immune self-tolerance. Overt autoimmunity due to viral infection is a well-recognized phenomenon^40^ that has also been shown with COVID-19^41^. Immunization is reported to reduce the likelihood of developing COVID-19-triggered autoimmune disease^42^, but there are various publications showing that some COVID-19 vaccines could increase risks for certain autoimmune states^4–6^. In the context of antibody-mediated AEFIs, VITT remains the best-understood thrombotic AEFI, and is caused by presence of autoantibodies to PF4-heparin complexes among individuals receiving COVID-19 vaccines^21–25^. In addition to clinical research associating autoantibodies against PF4/PF4C with catastrophic thrombosis, it has also been revealed that these responses are novel and do not emerge as a result of natural or vaccine-induced SARS-CoV-2 immunoreactivity^43^. Notably, similar autoantibodies to PF4 have recently been shown to manifest after adenoviral infections, described as VITT-like antibodies^44–46^. A structural analysis of PF4 autoantibodies with these different origins (post-vaccination and post-adenoviral infection) has demonstrated striking similarities between the antibodies, strongly suggesting that they are likely to be triggered by adenoviral exposure^47^. Our analyses did not reveal any patients with positivity for PF4 or PF4C autoantibodies. This was not unforeseen since VITT is extremely rare, with available data suggesting a frequency ranging between 1:26000 and 1:260000^22,48^.

Genetic deficiencies in Factor V, prothrombin, antithrombin III, protein S, and protein C are collectively known as hereditary (inherited) thrombophilias^49^. We hypothesized that autoantibodies neutralizing these proteins could underlie coagulation-related AEFIs, much like phenocopies of these diseases (eg, acquired protein S deficiency)^50^. Protein S is a crucial physiological anticoagulant that functions as a cofactor for activated protein C, thereby enhancing the inactivation of factors Va and VIIIa and resulting in downregulation of prothrombinase activity and clot formation^51^. The presence of neutralizing/blocking antibodies to this protein can favor the activation of the coagulation cascade, potentially tilting the balance towards procoagulant outcomes^50,52,53^. Pre-pandemic literature on this topic has shown a relationship between viral infections and protein S and/or C deficiency among patients suffering from post-viral thrombotic complications^13^. Indeed, deficiencies in protein S and C have also been associated with coagulopathy among patients with COVID-19^8–10^. Our data revealed 6 samples that could be deemed positive for protein S autoantibodies based on strict criteria. Four had coagulation-related AEFIs (thrombophlebitis, thrombocytopenia, pulmonary embolism, and pulmonary embolism + cerebral infarct) while two had other AEFIs (Guillain-Barré and vestibular neuritis). Notably, the two patients who had low protein S activity, as well as meeting positivity criteria, were diagnosed with thrombophlebitis and pulmonary embolism (coagulation-related AEFI). Prior literature supports our conclusions regarding the pathophysiological impact of these autoantibodies, as shown by direct relationships between protein S concentration, its impact on coagulation/thrombosis, and autoantibody levels^50,52–54^.

Circulating protein C levels and activity are reportedly decreased among patients who experience severe COVID-19 infection^55^. In addition, protein C expression is downregulated in the tissues of severe COVID-19 patients with thromboembolic complications^9^. As a critical contributor to protein C activity, antibody-mediated protein S dysfunction could be among the factors that explain the emergence of a procoagulant state. The literature on this topic has largely focused on reduced production of protein C despite the fact that functional activity is not always in proportion with protein concentration^56^. In fact, in contrast to studies showing decreased protein C among severe COVID-19 patients, there are reports that have shown high protein C but low protein S^57^. In this context, the presence of likely-persistent autoantibodies targeting protein S could potentially elucidate the underlying pathophysiology of rare but severe AEFIs associated with COVID-19 vaccines. It is also crucial to note that a disarray in the protein C and S system not only disrupts coagulation and increases thromboembolic risks, but may also lead to neurological adverse outcomes^58^. Both patients with non-coagulation AEFIs who were positive for protein S autoantibodies in our cohort had experienced neurological AEFIs.

During the early phases of the pandemic, numerous studies explored pro- and anti-coagulants in order to understand their potential roles in COVID-19-associated coagulopathy. Antiphospholipid autoantibodies became a primary focus in this regard due to their established association with viral infections^16,17^. Certain types of antiphospholipid autoantibodies were detected in up to 66% of patients with COVID-19, as reported by a meta-analysis which, despite showing high percentages of positivity for almost all antigens, did not detect any significant relationships with thrombotic events or disease severity^59^. Marginal research suggesting frequencies of over 80% (especially for lupus anticoagulant) among critically-ill patients, or those with prolonged aPTT, also exist^60–62^. Furthermore, a comprehensive study exploring multiple antibody targets ranked anti-cardiolipin and anti-platelet glycoprotein autoantibodies as the leading factors that could be associated with COVID-19 severity, which lends credibility to their possible contribution to hypercoagulability^63^. In a study specifically focusing on COVID-19 patients with thromboembolic events, it was suggested that these antibodies could indeed contribute to COVID-19-associated coagulopathy, but the authors emphasized that antiphospholipid antibodies alone were unlikely to cause an appreciable increase in event risk^18^, which is a view shared by other researchers^19,20^. We detected only two patients that could be classified as being positive for antiphospholipid antibodies, neither of whom had experienced coagulation-related AEFI. Considering the frequency of these antibodies in the general population (1 to 10%)^64^, our findings indicate that antiphospholipid antibodies are unlikely to be major contributors to AEFIs linked with COVID-19 vaccination.

Another aspect to consider in the interpretation of our results is the possible impact of patient-related characteristics on the development of AEFIs, particularly coagulation-related AEFIs. Although we employed WHO causality criteria^31^ to exclude patients for which AEFIs could be directly explained by factors other than vaccination, the older age of the coagulation-related AEFI group and the potential heterogeneities introduced by this difference deserve mention. In both subsets of our study, we found that the coagulation-related AEFI group was significantly older compared to other groups –except for the COVID-19-exposed group in the second subset. Elderly patients have a higher likelihood of having comorbidities, using chronic medications, and worse general condition, which could bias the analyses due to the impact of possible confounders that were not recorded. However, as mentioned previously, subjects with evidence for other causes were excluded from our AEFI cohort and our study focused on autoantibodies that could cause dysfunctions explaining coagulation-related events, which improves the reliability of our data in this context.

## Limitations

Despite casting a population-wide net, there is a possibility for bias associated with patient recruitment since physicians may have been more likely to diagnose AEFI among patients with relatively overt symptomatology that could be readily associated with vaccination. Additionally, patients who had catastrophic AEFIs resulting in mortality would be absent from the dataset due to the recruitment design of the SWEDEGENE study. Both of which can limit generalizability. As mentioned above, the older age of the coagulation-related AEFI group must be considered in terms of generalizability when examining our results. Secondly, these patients were not examined in the pre-pandemic period and longitudinal follow-up was not performed. The absence of this data and the delay between events and sampling make it impossible to draw definitive conclusions regarding the relationship between immunization and the emergence or pre-existence of autoantibodies. Laboratory testing for antiphospholipid antibodies necessitates at least two positive detections separated by 12 weeks, and therefore, the two cases identified in our cohort are not diagnostically relevant. Finally, the extended period between events and sampling could have introduced several types of bias as other vaccines (eg, influenza, hepatitis B etc.) might have been received in the interval or other events impacting immunoreactivity could have occurred. In the same context, the delay in blood collection precluded the detection of transient autoantibodies that could have emerged in the early period following vaccination; however, our focus was to investigate autoantibodies that were present for extended time periods since these would have been more likely to have pathological consequences.

## Conclusion

Leveraging population-wide data from the SWEDEGENE study, we examined long-standing immunoreactivity towards various coagulation-related proteins among patients who had experienced AEFIs attributed to COVID-19 vaccines. The results showed rare instances of autoantibodies against protein S in patients who had suffered from coagulation-related AEFIs (n = 4), with two patients in this group having evidence of functional impact on coagulation, likely explaining the pathophysiology of these select cases. The remaining patients with protein S autoantibodies did not have functional protein S impairment; however, this may have been associated with decreased antibody levels at the time of sampling relative to the levels coinciding with the event or vaccination, limiting the detection of their impact on protein S activity. We did not identify autoantibodies targeting PF4 or PF4-polyanion complexes, evidencing the extreme rarity of VITT, and there were also very few cases with antiphospholipid antibodies. Further studies stratifying patients and vaccines according to different characteristics are necessary to understand other factors that could be associated with the loss of self-tolerance after receiving COVID-19 vaccines, guiding the development of safer vaccines.

## Supplementary Information

Supplementary Information.

## Data Availability

The data that support the findings of this study and all code used for analyses are available from the corresponding authors upon reasonable request.
