# Supplementary material for: Autoantibodies to protein S may explain rare cases of coagulopathy following COVID-19 vaccination
Source: Sci Rep. 2024 Oct 18;14:24512. doi: 10.1038/s41598-024-75514-x (PMC11489816; doi:10.1038/s41598-024-75514-x)
Supplement: Supplementary file 1 — Supplementary Information. [file 41598_2024_75514_MOESM1_ESM.docx]

**SUPPLEMENTARY FILE**

**Autoantibodies to protein S may explain rare cases of coagulopathy following COVID-19 vaccination**

Ahmet Yalcinkaya^1, 2*^, Marco Cavalli^3, 4^, Maribel Aranda-Guillén^5^, Axel Cederholm^1^, Almira Güner^1^, Isabel Rietrae^1^, Hedvig Mildner^1^, Anish Behere^1^, Oskar Eriksson^6^, Laura Gonzalez^7^, Constantin Habimana Mugabo^7^, Anette Johnsson^7^, Tadepally Lakshmikanth^7^, Petter Brodin^7, 8^, Mia Wadelius^4^, Pär Hallberg^4^, Nils Landegren^1, 5^

^1^ Science for Life Laboratory, Department of Medical Biochemistry and Microbiology, Uppsala University, Uppsala, Sweden

^2^ Department of Medical Biochemistry, Faculty of Medicine, Hacettepe University, Ankara, Turkey

^3^ Science for Life Laboratory, Department of Immunology, Genetics and Pathology, Uppsala University, Uppsala, Sweden

^4^ Science for Life Laboratory, Clinical Pharmacogenomics, Department of Medical Sciences, Uppsala University, Uppsala, Sweden

^5^ Center for Molecular Medicine, Department of Medicine (Solna), Karolinska Institutet, Stockholm, Sweden

^6^ Department of Immunology, Genetics and Pathology, Uppsala University, Uppsala, Sweden

^7^ Unit for Clinical Pediatrics, Department of Women’s and Children’s Health (Solna), Karolinska Institutet, Stockholm, Sweden

^8^ Department of Immunology & Inflammation, Imperial College London, London, United Kingdom.

**Correspondence:** Ahmet Yalcinkaya, Department of Medical Biochemistry and Microbiology, Science for Life Laboratory, Uppsala University, Uppsala, Sweden; e-mail: [ahmet.yalcinkaya@imbim.uu.se](mailto:ahmet.yalcinkaya@imbim.uu.se)

| **Supplementary Table 1.** Diagnoses defined for the coagulation-related and other AEFI groups | | | |
| --- | --- | --- | --- |
| **Coagulation-related adverse events following immunization** | **Other adverse events following immunization** | | |
|  | **Neurologic** | **Allergic** | **Cardiac** |
| Amaurosis fugax | Abducens palsy | Allergic reaction | Myocarditis |
| Arterial thrombosis | Anosmia | Anaphylactic shock | Pericarditis |
| Hematoma | Numbness | Anaphylactic reaction | Perimyocarditis |
| Bleeding | Encephalitis | Angioedema |  |
| Cerebral sinus thrombosis | Encephalomyelitis | Urticaria |  |
| Deep venous thrombosis | Facial palsy |  |  |
| Ecchymosis | Guillain-Barré syndrome |  |  |
| Epistaxis | Hearing loss |  |  |
| Hemoptysis | Sensory disturbance |  |  |
| Pulmonary embolism | Smell disorder (parosmia) |  |  |
| Mesenteric vein thrombosis | Meningitis |  |  |
| Petechiae | Myelitis |  |  |
| Retinal hemorrhage | Myelopathy |  |  |
| Retinal artery occlusion | Neuropathy |  |  |
| Retinal artery thrombosis | Paresthesia |  |  |
| Retinal vein thrombosis | Polyneuropathy |  |  |
| Sinus thrombosis | Taste disorder (dysgeusia) |  |  |
| Subarachnoid hemorrhage | Vocal cord paralysis |  |  |
| Thrombophlebitis | Trochlear nerve paralysis |  |  |
| Thrombosis | Vestibular neuritis |  |  |
| Venous thrombosis | Dizziness |  |  |

**Supplementary Figure 1.** ELISA re-analysis of samples with elevated response to the protein S antigen in the bead-based assay

The “confirmation group” included the 6 samples defined to be positive based on bead-based assay criteria. The ELISA also included 8 samples from patients with myocarditis and 8 samples from the blood donor group (randomly selected). The positive control antibody was used at a concentration of 0.04 µg/ml.

**Supplementary methods**

***Immunoglobulin G purification from heparinized samples***

We utilized a mixture of protein A and protein G magnetic beads (Dynabeads, Invitrogen) to purify IgG from each heparinized plasma sample that was to be included in functional testing (8 patients with highest immunoreactivity to protein S). Briefly, equal volumes of protein A and protein G magnetic beads (Dynabeads, Invitrogen) were mixed and 25 ul of the bead suspension mix was pipetted into 1.5-ml eppendorfs, followed by two washes with PBS containing 0.02% Tween-20. The washes were performed by adding 500 ul of wash buffer, vortexing, magnetizing, and removing the supernatant. In the next step, 100 ul of binding buffer (PBST 0.02%) and 50 ul of plasma sample were added in succession to the washed beads. The eppendorfs were mixed thoroughly and incubation was performed with end-over-end mixing on a tilting mixer for 40 minutes at 4°C. When the incubation to bind IgGs was complete, the eppendorfs were magnetized and the supernatant was removed during the course of three washes. The bead-bound IgG was eluted using 0.2 molar glycine adjusted to a pH of 2.5 with HCl (elution buffer). The beads were mixed with 25 ul of elution buffer, vortexed and left for 5 minutes of end-over-end mixing at room temperature. Eluents were collected following magnetization and a second elution step was performed to ensure maximum purification efficiency. The two eluents from each sample were pooled in fresh eppendorfs, creating 45-50 ul of purified IgG, which was neutralized by adding 10 ul of 1 molar Tris-HCl (adjusted to a pH of 9.0 with HCl).

***ACTICLOT Protein S assay***

A 50 ul volume of purified IgG was mixed with 50 ul of pooled plasma (EDTA plasma from 15 subjects). This mixing caused an initial 1:2 dilution of IgG and pooled plasma. Since the ACTICLOT assay was designed for 1:10 dilution, these specific samples were diluted 1:5 during the assay to obtain correct dilution of IgG. All other steps of the analysis were performed based on manufacturer instructions, except for the timing for clot development which was performed manually. A standard curve for clotting time was created with analysis of pooled plasma samples (100% plasma, 50% plasma, and 0%–dilution buffer). Shorter clotting time indicates lower protein S activity, while longer clotting time indicates higher protein S activity. Reference range for normal protein S activity is reported as being 55% to 160% of pooled plasma results. All four blood donors tested were within this reference range (85.7%–128%).
